# Pathway crosstalk enables degradation of aromatic compounds in marine *Roseobacter* clade bacteria

**DOI:** 10.1128/aem.00978-25

**Published:** 2025-08-12

**Authors:** Huan-Wei Xu, Xiao-Yan Wang, Ying Wei, Yiqi Cao, Shu-Guang Wang, Peng-Fei Xia

**Affiliations:** 1 School of Environmental Science and Engineering, Shandong University, Qingdao, China; 2 Northern Region Persistent Organic Pollution Control (NRPOP) Laboratory, Faculty of Engineering and Applied Science, Memorial University 7512, St. John's, Canada; 3 School of Marine Sciences, Ningbo University, Ningbo, China; 4 Sino-French Research Institute for Ecology and Environment, Shandong University, Qingdao, China; 5 Weihai Research Institute of Industrial Technology, Shandong University, Weihai, China; Universidad de los Andes, Bogotá, Colombia

**Keywords:** aromatic compounds, *Roseobacter *clade, marine bacteria, β-ketoadipate pathway, multi-omics, CRISPR-Cas

## Abstract

Aromatic compounds are essential raw materials for almost all sectors of human societies but also persistent environmental pollutants recalcitrant to biodegradation. The ocean serves as a significant sink for these compounds, while their biological conversion routes remain poorly understood, hindering a comprehensive understanding of the marine carbon cycle and advancements in bioremediation and biological carbon upcycling. Here, we report the degradation pathway of aromatic molecules in the marine *Roseobacter* clade bacteria through multi-omics analysis and CRISPR-Cas-based genome editing. Using *Roseovarius nubinhibens* and 4-hydroxybenzoate (4HB) as representatives, we identified the transport of 4HB via TRAP, ABC, and MFS transporters. Then, we deciphered the integral β-ketoadipate pathway responsible for aromatic degradation. Next, we discovered a distinct pathway crosstalk at the final thiolation step, which serves as an intersection node of different pathways catalyzed by the 3-oxoadipyl-CoA thiolase from the β-ketoadipate pathway and the acetyl-CoA C-acetyltransferase and acetyl-CoA C-acyltransferase from the β-oxidation pathway. Finally, we proposed *R. nubinhibens* as a novel marine platform for systems-level interrogation and bioprospecting. Our study provides a foundation for leveraging *Roseobacter* clade bacteria as novel chassis for environmental and industrial innovations.

**IMPORTANCE:**

Aromatic compounds lie in an essential node of carbon cycling in both natural and engineered systems. Marine bacteria orchestrate the cycling of aromatic compounds in the ocean and, as emerging chassis, have shown unusual potentials in the degradation and valorization of aromatics. However, the corresponding metabolic pathway in marine bacteria remains poorly interpreted over decades, hindering further scientific interrogation and engineering practices. Here, we deciphered the complete degradation pathway of aromatic compounds in the marine *Roseobacter* clade bacteria and established a marine platform for systems and synthetic biology. Our study provides a paradigm for biological interrogation with combined multi-omics and the cutting-edge CRISPR-Cas approaches, laying a foundation for biological innovations with marine bacteria.

## INTRODUCTION

Aromatics are ubiquitous and abundant carbon compounds on the planet. These compounds are essential for all living organisms as amino acids and secondary metabolites (1), constitute approximately one-quarter of the plant biomass on Earth as the building blocks of lignin (2), and are key components of crude oil, providing raw materials (e.g., benzene, toluene, and xylene) for human societies (3). However, aromatics are also common environmental contaminants. Their structural stability and diversity make aromatic pollutants, such as polycyclic aromatic hydrocarbons (PAHs) and polystyrene plastic, highly resistant to degradation, especially biological conversions (4–6). Moreover, conventional disposal approaches for lignin, such as incineration, lead to significant emissions of CO_2_, NO_x_, and inhalable particles (7–9). Aromatic compounds synergize a critical nexus connecting bioremediation, resource upcycling, and biomanufacturing, necessitating more efforts in interrogating the biological fundamentals and advancing technological innovations.

Marine *Roseobacter* clade bacteria comprise approximately 15–25% of the total oceanic bacterial community and play important roles in maintaining marine ecosystems and driving biogeochemical cycles (10, 11). Bacteria in this clade usually harbor versatile metabolic capabilities, including anoxygenic photosynthesis, carbon monoxide oxidation, dimethylsulfoniopropionate demethylation, and the degradation of recalcitrant organic compounds, for instance, aromatics (12–17). Some marine *Roseobacter* clade bacteria can efficiently metabolize aromatic compounds through the β-ketoadipate pathway (13, 18), and strains isolated from environments contaminated by PAHs and oils are capable of degrading fluoranthene and pyrene (19), persistent pollutants with fused benzene rings. The unique metabolic feature has been leveraged for upcycling lignin-derived monomers into pharmaceutics by reprogramming the carbon flux in a marine *Roseobacter* clade bacterium, *Roseovarius nubinhibens*, providing a novel microbial chassis (20).

The degradation pathways of aromatic compounds in *Roseobacter* clade bacteria, unfortunately, remain poorly interpreted. Early studies identified six genes encoding the essential enzymes in the β-ketoadipate pathway, including *pobA*, *pcaH*, *pcaG*, *pcaC*, and *pcaD* as an operon led by *pcaQ* as the transcription activator, with *pcaF* located at a distinct locus in the genome (18, 21). All these genes were preliminarily identified through sequence alignment without experimental validation at the genetics level until a previous report confirmed the essentiality of *pobA*, *pcaHG*, and *pcaQ* using CRISPR-Cas-based genome editing approaches (22). However, the pathway remains incomplete for over 20 years, and the systems-level responses to aromatic substrates, such as the interactions between the β-ketoadipate and other pathways, have not been elucidated, leaving the pathway isolated and hardly integrated into the metabolic networks. This incomplete understanding significantly hinders advancements in the efficient degradation and upcycling of aromatic compounds and a thorough evaluation of the carbon cycle in the ocean.

Here, we systematically interrogated the aromatic compound metabolism in marine *Roseobacter* clade through combined multi-omics analysis and CRISPR-Cas-based genetic perturbation. Taking *R. nubinhibens* and 4-hydroxybenzoate (4HB) as representatives, we first evaluated the transport of 4HB. Then, we successfully identified and validated the previously unidentified essential genes in the β-ketoadipate pathway. Next, we uncovered the distinct crosstalk between the β-ketoadipate and β-oxidation pathways which connects these metabolic routes to the tricarboxylic acid (TCA) cycle, eventually allowing the degradation of aromatics. This is the first report illustrating the integral β-ketoadipate pathway in marine *Roseobacter* clade bacteria with genetic evidence, shedding light on the conversion of aromatic compounds under natural and engineered contexts.

## RESULTS

### Multi-omics landscape of *R. nubinhibens* growing with 4HB

**Fig 1:**
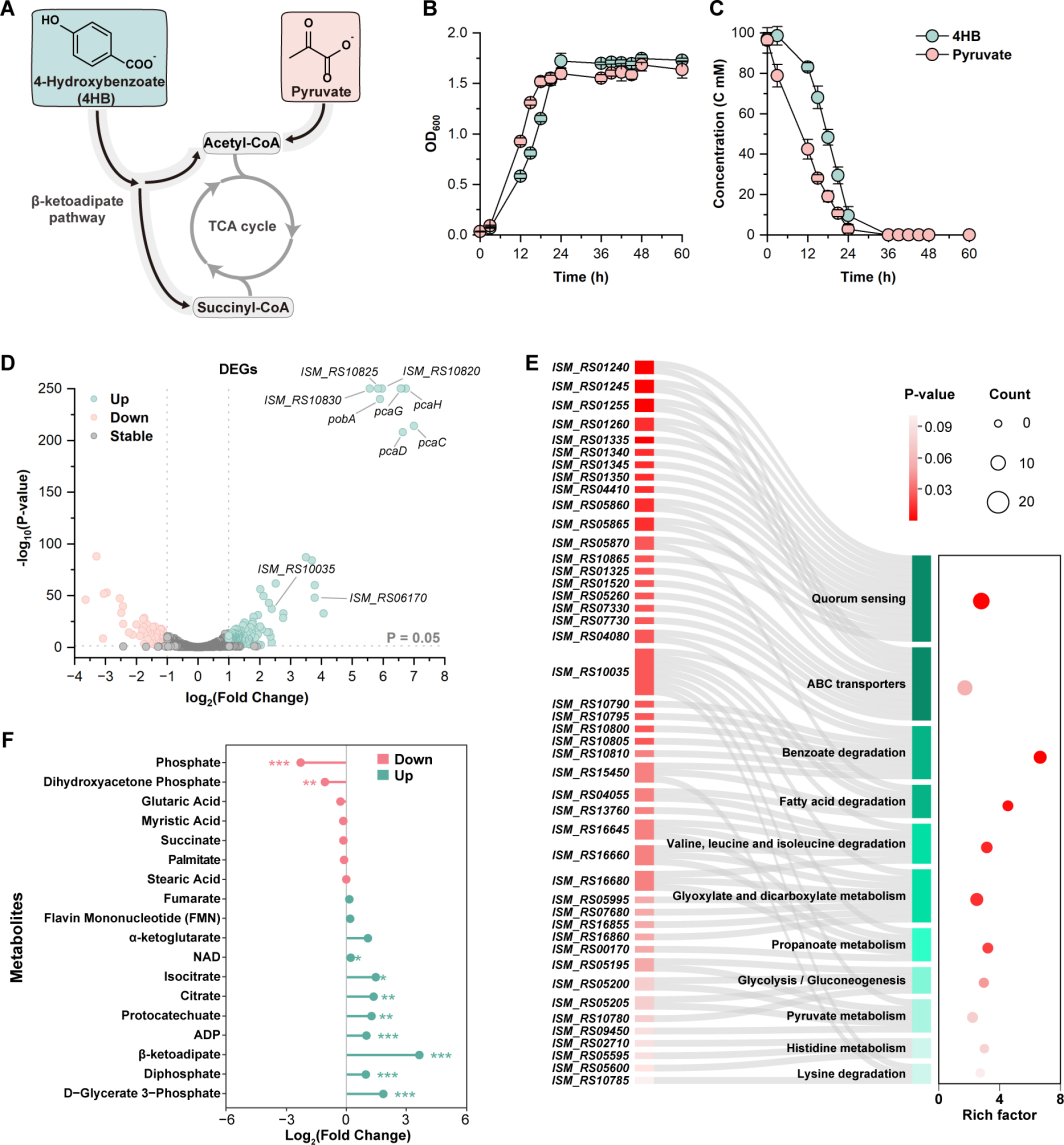
Overview of multi-omics analysis of *R. nubinhibens* with 4HB as the carbon source. (**A**) Schematic representation of 4HB and pyruvate metabolism into the TCA cycle. In *R. nubinhibens*, 4HB is converted to acetyl-CoA and succinyl-CoA through β-ketoadipate pathway into the TCA cycle, while pyruvate can be directly converted to acetyl-CoA. (**B**) Growth profiles for *R. nubinhibens*. (**C**) Consumption of 4HB and pyruvate. The wild-type *R. nubinhibens* strain was cultivated in MBM with 4HB and pyruvate as carbon sources. The two substrates were added at the same molar concentration of carbon. OD_600_ and substrate concentrations were measured at different time intervals. The experiments were performed in triplicate, and error bars represent the standard deviations. (**D**) Volcano plot showing the fold change in gene expression. Upregulated differentially expressed genes (DEGs) were identified with log_2_ (fold change) >1 and *P*-value < 0.05, highlighted in green. Downregulated DEGs were identified with log_2_ (fold change) < −1 and *P*-value < 0.05, highlighted in pink. Stably expressed genes are marked in gray. (**E**) KEGG pathway enrichment analysis on DEGs. The circle size represents the number of enriched genes in each pathway, and the gradient red in the circle represents the *P*-value. Different shades of red and green rectangles indicate different genes and metabolic pathways, respectively. (**F**) Lollipop map of metabolites. Metabolites with log_2_ (fold change) >0 are shown in green, while <0 are shown in pink. Statistical significance was determined using *t-*test (*P < 0.05; **P < 0.01; ***P < 0.001).

To investigate the aromatic metabolism pathway, we chose 4HB as the representative aromatic compound, which is degraded by *R. nubinhibens* through β-ketoadipate pathway, and used pyruvate as the control substrate. Pyruvate enters the TCA cycle with acetyl-CoA as the sole intermediate, thereby minimizing the influences of other pathways (Fig. 1A). *R. nubinhibens* exhibited similar growth profiles in marine basal medium (MBM) with 4HB and pyruvate as carbon sources, which were standardized to the same molar concentration of carbon (Fig. 1B and C). Additionally, we ruled out the potential impacts of pH, an important environmental factor that induces systems-level responses in *R. nubinhibens*. Starting from an initial pH of 7.5, the final pH values were similar and reached around 8.5–9.0 with these two distinct carbon sources (Fig. S1), agreeing with our previous observation (23).

We performed transcriptomic and proteomic analyses on *R. nubinhibens* at the exponential phase. Out of all 3,532 genes, we identified 177 upregulated DEGs and 97 downregulated DEGs for the strain growing with 4HB compared to pyruvate (Fig. 1D). The transcriptomic data were further validated using quantitative real-time PCR (RT-qPCR) (Fig. S2). The proteomic analysis determined 244 upregulated differentially expressed proteins (DEPs) and 148 downregulated DEPs (Fig. S3). KEGG pathway enrichment analysis of upregulated DEGs and DEPs revealed significant enrichment in pathways associated with benzoate degradation, glyoxylate and dicarboxylate metabolism, and fatty acid degradation at both mRNA and protein levels. Notably, all genes of the *pca* gene cluster, which encodes the key enzymes involved in the β-ketoadipate pathway, exhibited highly significant upregulation (Fig. 1D). Additionally, we found that the enzyme ADP-forming succinate-CoA ligase in the TCA cycle was significantly upregulated in the presence of 4HB (Table S1), while no significant changes were identified for other enzymes in the TCA cycle. Interestingly, pyruvate metabolism, ABC transporter, and quorum sensing exhibited contrary enrichment results at the mRNA and protein levels, indicating uncovered regulation mechanisms at transcription and translation levels (Fig. 1E; Fig. S4). For instance, five upregulated DEGs were enriched in the pyruvate metabolism, while the four corresponding proteins were downregulated (Fig. S5).

Metabolomic analysis identified 303 upregulated differentially expressed metabolites (DEMs) and 380 downregulated DEMs from 2,817 detected metabolites (Fig. S6). Consistent with the analysis above, the key intermediates of the benzoate degradation pathway, protocatechuate and β-ketoadipate, were upregulated by 2.40-fold and 12.60-fold, respectively. In contrast, metabolites involved in fatty acid degradation and biosynthesis (e.g., glutaric acid, myristic acid, palmitate, and stearic acid) showed no significant changes, and no significant DEMs were associated with pyruvate metabolism. For the TCA cycle, citrate, isocitrate, and α-ketoglutarate were upregulated by 2.58-fold, 2.78-fold, and 2.11-fold, respectively. Additionally, energy- and redox-related metabolites exhibited a varied dynamic. Specifically, ADP and diphosphate increased by 2.00-fold and 1.97-fold, while flavin mononucleotide (FMN) and NAD remained unchanged (Fig. 1F). The omics analysis highlighted the essential role of the β-ketoadipate pathway in 4HB metabolism, and multiple metabolic pathways might respond to the changed substrate.

### The transport of 4HB relies on TRAP, ABC, and MFS transporters

**Fig 2:**
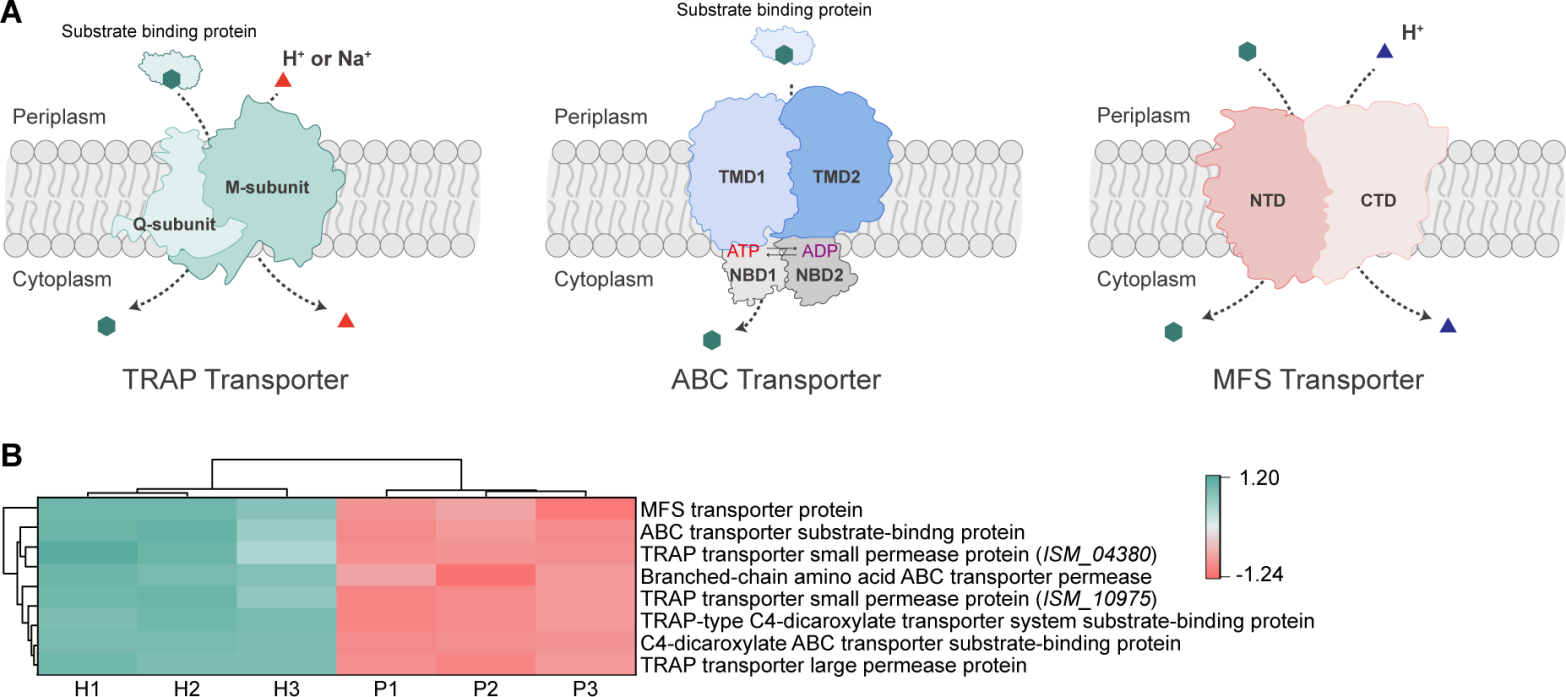
The transport of 4HB in *R. nubinhibens***.** (**A**) Schematic representation of 4HB uptake in *R. nubinhibens* mediated by ABC, TRAP, and MFS transporters. Regular hexagons in dark green represent the 4HB, and triangles represent ions (H^+^ or Na^+^). TMD, transmembrane domain; NBD, nucleotide-binding domain; NTD, N-term six TM-bundle; CTD, C-term six TM-bundle. TRAP transporter consists of a substrate binding protein and two transmembrane domains, Q-subunit and M-subunit, and relies on a gradient generated by Na^+^ or H^+^ for unidirectional transmembrane transport. ABC transporter, consisting of a substrate-binding protein, two nucleotide-binding domains, and two transmembrane domains, enables substrate transport across the membrane by consuming ATP. MFS transporter achieves transmembrane transport of substrates by utilizing proton dynamics on both sides of the membrane. This process requires the electrochemical gradient of cations (e.g., H^+^) to provide energy for transport. (**B**) Heatmap of proteins related to the three transporters. H and *P* represent 4HB and pyruvate groups, respectively.

The active transport of aromatic compounds primarily depends on the tripartite ATP-independent periplasmic (TRAP) transporter, the ATP-binding cassette (ABC) transporter, and the major facilitator superfamily (MFS) transporter (Fig. 2A) (24). We found that the clustered *ISM_RS10820*, *ISM_RS10825*, and *ISM_RS10830*, encoding the TRAP transporter proteins, were significantly upregulated (Fig. 1D), which was in agreement with the upregulated TRAP transporter proteins revealed by proteomics (Fig. 2B). Notably, these genes are located 159 bp upstream of the *pca* gene cluster, suggesting a potential linkage with the β-ketoadipate pathway (Fig. S7). Additionally, the function of TRAP transporters has been demonstrated in another *Roseobacter* clade bacterium *Sagittula stellata* E-37 (25). Our data also revealed the involvement of ABC and MFS transporters in 4HB transport. Pathways associated with ABC transporters were significantly enriched, with all 11 genes enriched in this pathway encoding the ABC transporter-related proteins (Fig. 1E). Moreover, several ABC transporter-associated proteins exhibited significant upregulation at the protein level (Fig. 2B). In addition, five genes (*ISM_RS17060*, *ISM_RS08735*, *ISM_RS15525*, *ISM_RS13825*, and *ISM_RS16685*) encoding MFS transporters were upregulated at the mRNA level, with *ISM_RS15525* showing significant upregulation at the protein level (Fig. S8; Fig. 2B). According to the literature, ABC transporters and MFS transporters have shown important roles in the transport of aromatics in diverse environmental bacteria, including *Rhodopseudomonas palustris*, *Sphingobium* sp., and *Pseudomonas putida* (26–29). These data suggested that all three types of transporters might contribute to the uptake of 4HB in *R. nubinhibens*.

### Uncovering the missing genes in the β-ketoadipate pathway

**Fig 3:**
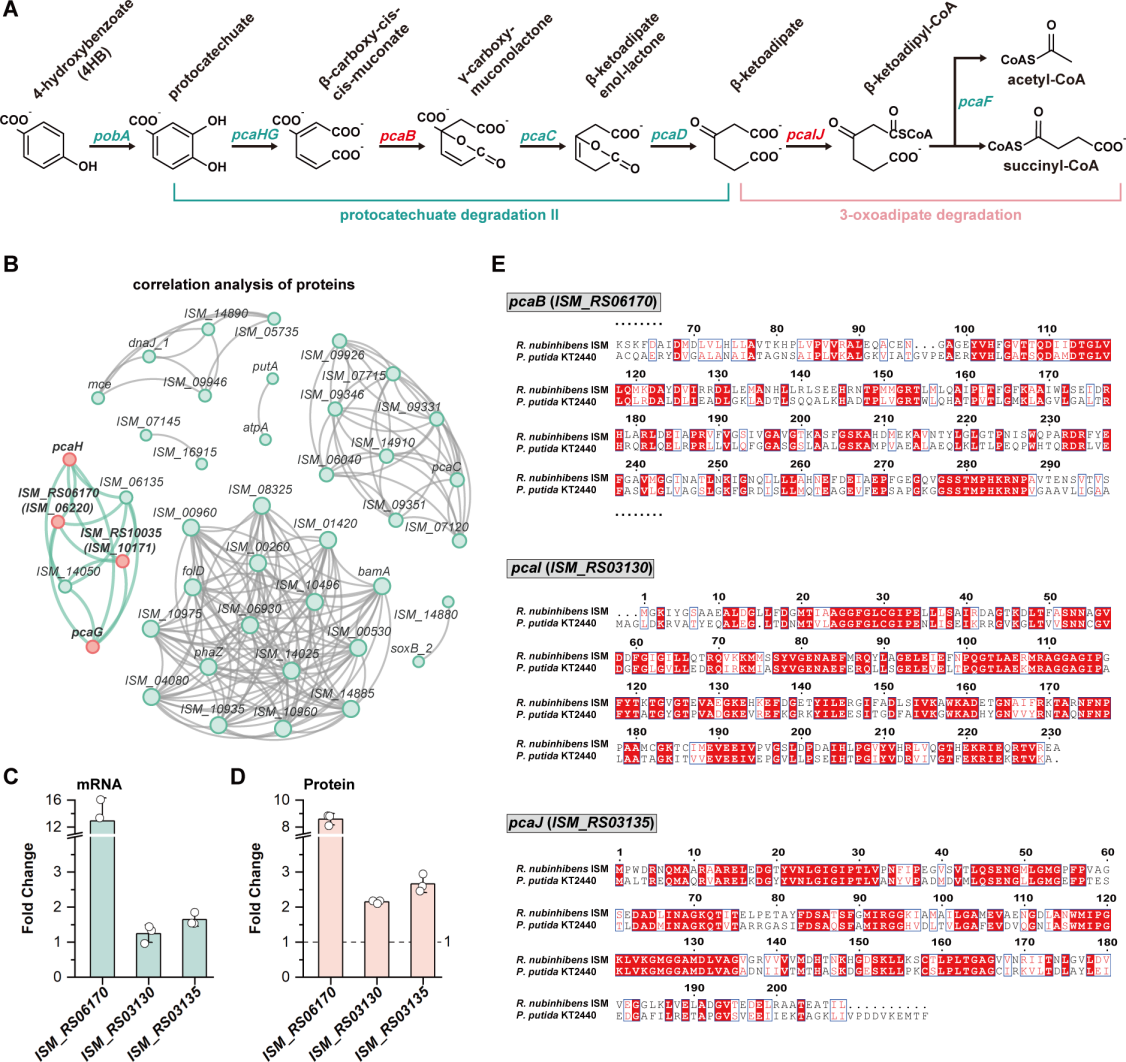
Identification of the unidentified genes in the β-ketoadipate pathway. (**A**) β-ketoadipate pathway in *R. nubinhibens*. Genes that have not yet been identified are highlighted in red. (**B**) The co-occurrence network of Spearman correlation analysis of upregulated DEPs. The corresponding proteins are labeled with the gene numbers. Samples located in the same region are considered to be strongly correlated with each other. Fold change of *ISM_RS06170*, *ISM_RS03130,* and *ISM_RS03135* at the (**C**) mRNA and (**D**) protein levels. (**E**) Visualization of alignment between the amino acid sequences of *pcaB*, *pcaI,* and *pcaJ* encoded by *R. nubinhibens* and *P. putida* KT2440. White letters with a red background indicate identical sequences. Red letters with a white background highlight non-identical residues with similar physicochemical properties.

The protocatechuate branch of the β-ketoadipate pathway serves as the primary route for the degradation of benzoates in *Roseobacter* clade bacteria. Contrary to the well-defined genes in the *pca* cluster, three genes, *pcaB*, *pcaI*, and *pcaJ*, remained unidentified. *pcaB* encodes the 3-carboxy-*cis,cis*-muconate cycloisomerase, while *pcaI* and *pcaJ,* usually adjointly located in an operon (30, 31), together encode the 3-oxoadipate CoA-transferase (Fig. 3A). To identify these missing genes, we analyzed the top 20 DEGs and performed Spearman rank correlation analysis on 50 DEPs (Fig. S9; Fig. 3B). We found that *ISM_RS06170* was significantly upregulated at the mRNA and protein levels, exhibiting strong protein-level correlations with *pcaH* and *pcaG*, encoding ring-cleavage protocatechuate 3,4-dioxygenase (Fig. 3B, C and D). The sequence alignment showed 36.16% and 27.67% identity between *ISM_RS06170* and *pcaB* from *P. putida* KT2440 and *Corynebacterium glutamicum* ATCC 13032, respectively (Fig. 3E; Fig. S10), making *ISM_RS06170* the *pcaB* candidate. Then, we attempted to identify *pcaIJ* from direct sequence alignment using the annotated genes from *P. putida* KT2440 and *C. glutamicum* ATCC 13032, and the adjoint *ISM_RS03130* and *ISM_RS03135* were identified as the candidates (Fig. 3E; Fig. S10). Moreover, we found that expressions of *ISM_RS03130* and *ISM_RS03135* were increased 1.24-fold and 1.64-fold at the transcription level and significantly upregulated at the protein level by 2.15-fold and 2.66-fold, respectively (Fig. 3C and D).

**Fig 4:**
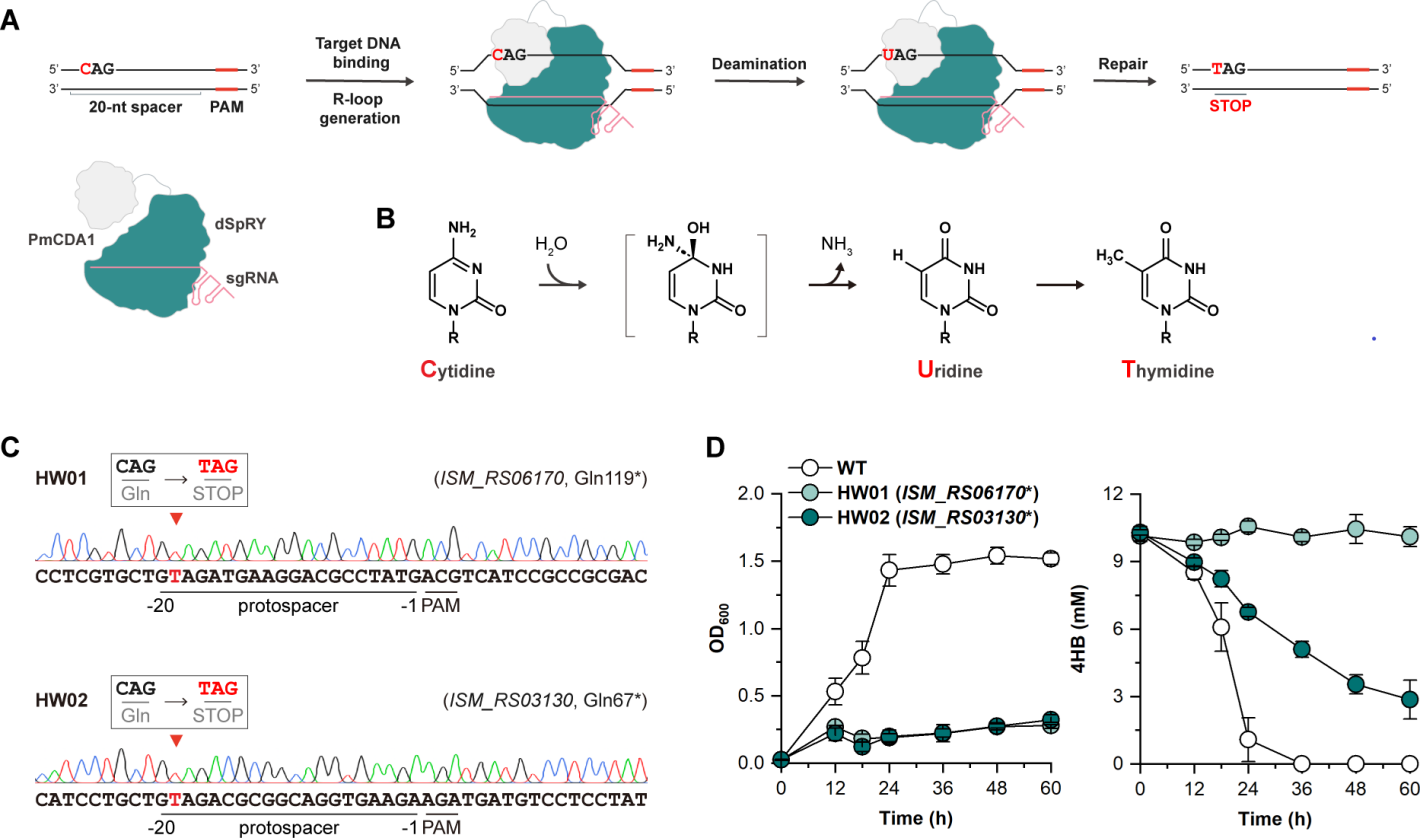
Verification of the unidentified genes using CRISPR-Cas base editing. (**A**) Schematic representation of gene inactivation via the base editing system. The CRISPR-Cas base editor binds to the target DNA and generates an R-loop. The deaminase PmCDA1 mediates the transition of cytidine (C) to uridine (U), which is subsequently changed to thymidine (T) through DNA repair. This C-to-T transition at the target site effectively transforms the glutamine-encoding CAG codon into a premature STOP codon (TAG), thereby inactivating a target gene. (**B**) The chemistry of deaminase-mediated C-to-T transition. (**C**) Sequencing results of base edited genes. The editing sites were indicated by red arrows. The edited bases were highlighted in red. (**D**) Growth profiles and 4HB utilization of the edited strains. Strains were cultivated in the MBM with 10 mM 4HB as the carbon source. OD_600_ and 4HB concentration were measured at different time intervals. All experiments were performed in triplicate, and error bars represent the standard deviations.

Next, we interrogated the functions of these newly identified genes at the genetics level. The degradation of 4HB will be eliminated or inhibited if these genes are deactivated. We employed a CRISPR-Cas base editing system established in our lab to inactivate corresponding genes. Leveraging a nuclease-deactivated Cas9 derivative dSpRY (32), a deaminase PmCDA1 from *Petromyzon marinus* (33), and a programmable guide RNA (gRNA), the base editing systems can target any loci in the genome of *R. nubinhibens* without dependence on the protospacer adjacent motif (PAM) and make cytosine (C) to thymine (T) editing (34, 35). By introducing the C-to-T transition at specific codons, the system can generate the premature STOP codon, thereby inactivating the target gene (Fig. 4A and B). We successfully constructed two edited strains with inactivated *ISM_RS06170* (HW01) and *ISM_RS03130* (HW02), respectively (Fig. 4C ; Table S2). As *ISM_RS03130* and *ISM_RS03135* together encode a functional 3-oxoacid CoA-transferase, the deactivation of *ISM_RS03130* alone should block the β-ketoadipate pathway. As expected, both deactivated strains could not grow with 4HB as the carbon source in MBM, while the slight growth might result from yeast extract in the medium. Notably, the *ISM_RS06170*-deficient strain HW01 failed to consume 4HB during cultivation, evidencing *ISM_RS06170* as *pcaB*. The *ISM_RS03130*-deficient strain HW02 exhibited severely repressed 4HB consumption (Fig. 4D). The consumption of 4HB might be attributed to the conversion of β-ketoadipate to other metabolites (21, 36). By integrating the omics analysis and genetic evidence, we can complete the β-ketoadipate pathway with *ISM_RS06170*, *ISM_RS03130*, and *ISM_RS03135* as *pcaB*, *pcaI*, and *pcaJ*, respectively.

### *pcaF* is not the predominant gene for the thiolysis

**Fig 5:**
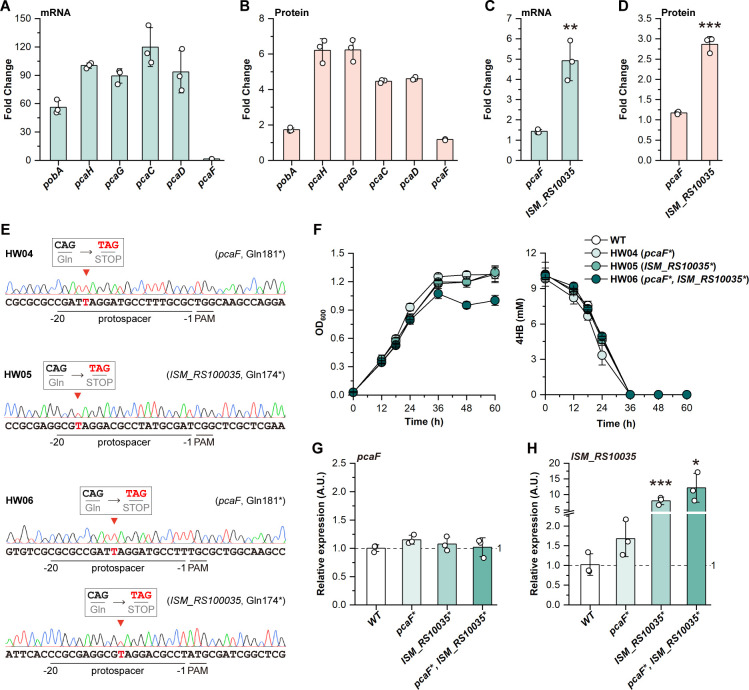
Functional validation of *pcaF* and *ISM_RS10035***.** Fold change of previously known genes (*pobA*, *pcaH*, *pcaG*, *pcaC*, *pcaD,* and *pcaF*) in the β-ketoadipate pathway at the (**A**) mRNA and (**B**) protein levels. Fold change of *pcaF* and *ISM_RS10035* at the (**C**) mRNA and (**D**) protein levels. (**E**) Sequencing results of the base-edited strains. The edited loci were indicated by red arrows. The edited bases were highlighted in red. (**F**) Growth profiles and 4HB utilization of the edited strains. (G) Relative expression of *pcaF* and (**H**) *ISM_RS10035* in the three edited strains, compared to the wild-type strain. All experiments were performed in triplicate, and error bars represent standard deviations. Statistical significance was determined using *t*-test (*P < 0.05; **P < 0.01; ***P < 0.001).

We observed that *pcaF,* encoding the 3-oxoadipyl-CoA thiolase*,* showed negligible response to the changed substrate, while all other genes in the β-ketoadipate pathway were significantly upregulated (Fig. 5A and B). This led us to assume that *pcaF* might not be the gene responsible for the thiolysis of β-ketoadipyl-CoA, and that other enzymes catalyze the conversion. Through a preliminary screening of the top 20 DEGs and the protein correlations (Fig. S9; Fig. 3B), we found that *ISM_RS10035*, encoding the acetyl-CoA C-acyltransferase (EC 2.3.1.16), was substantially upregulated, and its expression was significantly higher than *pcaF* at both the mRNA and protein levels (Fig. 5C and D). A 43.11% identity between *pcaF* and *ISM_RS10035* was confirmed via sequence alignment (Fig. S11), suggesting *ISM_RS10035 as* an alternative for *pcaF*.

To determine the roles at the genetics level, we deactivated *pcaF* and *ISM_RS10035*, respectively (Fig. 5E). Unexpectedly, there was no observable growth difference when comparing *pcaF* and *ISM_RS10035-*deficient strains to the wild-type, where 4HB was completely consumed at a similar rate within 36 h (Fig. 5F). Then, we deactivated both genes in one single strain (Fig. 5E), and the double-deficient strain exhibited a decreased final OD_600_ compared to the wild-type strain after 36-h cultivation (Fig. 5F). Furthermore, we analyzed the transcription levels of *pcaF* and *ISM_RS10035* in all the edited strains. The results showed that, compared to the wild-type, the transcription levels of *ISM_RS10035* increased by 1.68-fold, 7.96-fold, and 12.13-fold in *pcaF*-deficient, *ISM_RS10035*-deficient, and the *pcaF* and *ISM_RS10035* double-deficient strains, respectively, while no obvious differences could be identified for *pcaF* across the edited strains (Fig. 5G and H). A premature STOP codon generated by base editing inactivates a gene at the translation level, yet results in limited impacts on transcription. This feature offers a unique perspective to analyze the regulation of a gene. For instance, if *ISM_RS10035* was essential and actively regulated in the 4HB metabolism, the mRNA level might be significantly increased in the deficient strains due to continuous upregulation by the non-degraded substrates. Therefore, *pcaF* and *ISM_RS10035* indeed contributed to the degradation of 4HB but might be mutually complementary, and *ISM_RS10035*, rather than *pcaF*, exhibited a more active role in the pathway. However, the results suggested that other enzymes or metabolic routes can complement *pcaF* and *ISM_RS10035* for 4HB degradation.

### Pathway crosstalk enables 4HB degradation

**Fig 6:**
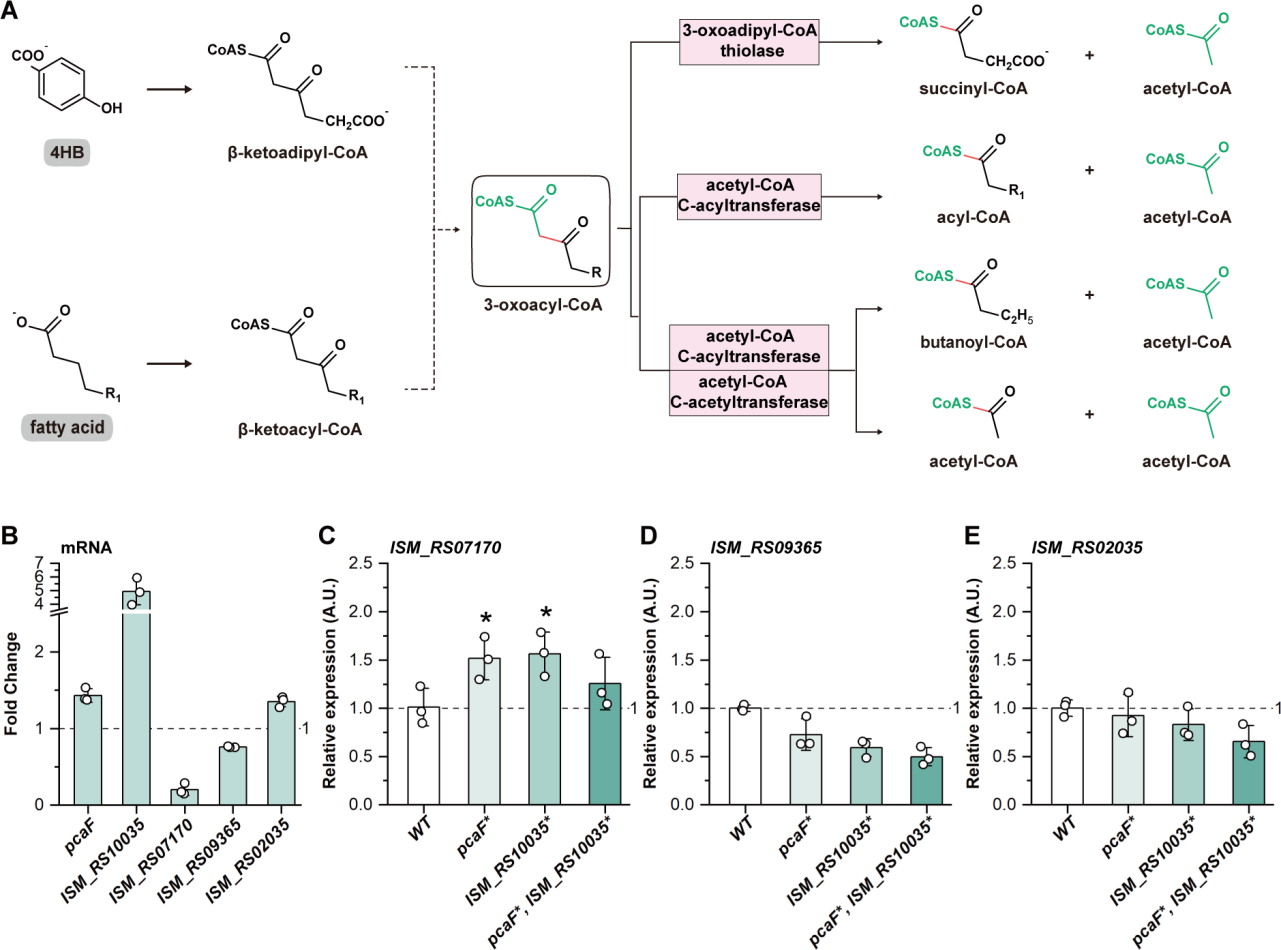
Crosstalk between the β-ketoadipate and β-oxidation pathways. (**A**) Schematic representation of the 3-oxoacyl-CoA catalytic pathways. The enzymes’ information is based on the Kyoto Encyclopedia of Genes and Genomes (KEGG) and BioCyc databases. In *R. nubinhibens*, 3-oxoadipyl-CoA thiolase (EC 2.3.1.174) and acetyl-CoA C-acyltransferase (EC 2.3.1.16) are encoded by *pcaF* and *ISM_RS10035,* respectively, and acetyl-CoA C-acetyltransferase (EC 2.3.1.9) is independently encoded by *ISM_RS07170*, *ISM_RS09365,* and *ISM_RS02035.* Chemical bonds cleaved by enzymatic reactions are highlighted in red. R and R_1_ denote distinct variable organic groups. (**B**) Fold change of the five genes (*pcaF*, *ISM_RS10035*, *ISM_RS07170*, *ISM_RS09365,* and *ISM_RS02035*) in wild-type strain at the mRNA level. (**C**) Relative expression of *ISM_RS07170*, (**D**) *ISM_RS09365*, and (**E**) *ISM_RS02035* in the three edited strains. All experiments were performed in triplicate, and error bars represent the standard deviations. Statistical significance was determined using *t-*test (*P < 0.05).

The acetyl-CoA C-acyltransferase (EC 2.3.1.16), encoded by *ISM_RS10035*, is primarily involved in the β-oxidation pathway—a metabolic route responsible for fatty acid degradation—catalyzing the cleavage of 3-oxoacyl-CoA to acetyl-CoA and a two-carbon shortened acyl-CoA (37, 38). Due to the structure of β-ketoadipyl-CoA, it is chemically rational that *ISM_RS10035* could complement *pcaF* (EC 2.3.1.174) (Fig. 6A). We noticed that another enzyme in the β-oxidation pathway, the acetyl-CoA C-acetyltransferase (EC 2.3.1.9), which can be independently encoded by *ISM_RS07170*, *ISM_RS09365,* and *ISM_RS02035* (Table S3), shares the similar catalytic activities to acetyl-CoA C-acyltransferase with a specific substrate preference on short-chain 3-oxoacyl-CoA (Fig. 6A). All these enzymes together might allow crosstalk between the β-oxidation and β-ketoadipate pathways, collaboratively achieving the degradation of 4HB.

The first evidence was that the β-oxidation pathway exhibited significant enrichment at both the mRNA and protein levels, while the metabolites showed negligible changes (Fig. 1E and F and Fig. S4). However, the transcriptomic analysis showed that *ISM_RS07170* was downregulated, and *ISM_RS09365* and *ISM_RS02035* showed no significant changes with fully functional *pcaF* and *ISM_RS10035* (Fig. 6B). Then, we performed RT-qPCR to determine the changes of these three genes coding for the acetyl-CoA C-acetyltransferase in the edited strains. The results showed that the expression of *ISM_RS07170* increased in *pcaF*, *ISM_RS10035*, and *pcaF* and *ISM_RS10035* double-deficient strains (Fig. 6C), while *ISM_RS09365* and *ISM_RS02035* showed decreased expression (Fig. 6D and E), demonstrating the compensating role mediated by *ISM_RS07170*. Therefore, the acetyl-CoA C-acyltransferase encoded by *ISM_RS10035* and acetyl-CoA C-acetyltransferase encoded by *ISM_RS07170* could compensate for the 3-oxoadipyl-CoA thiolase encoded by *pcaF*, allowing pathway-crosstalk–mediated degradation of aromatic compounds.

### Establishing a marine platform for systems-level interrogation and bioprospecting

**Fig 7:**
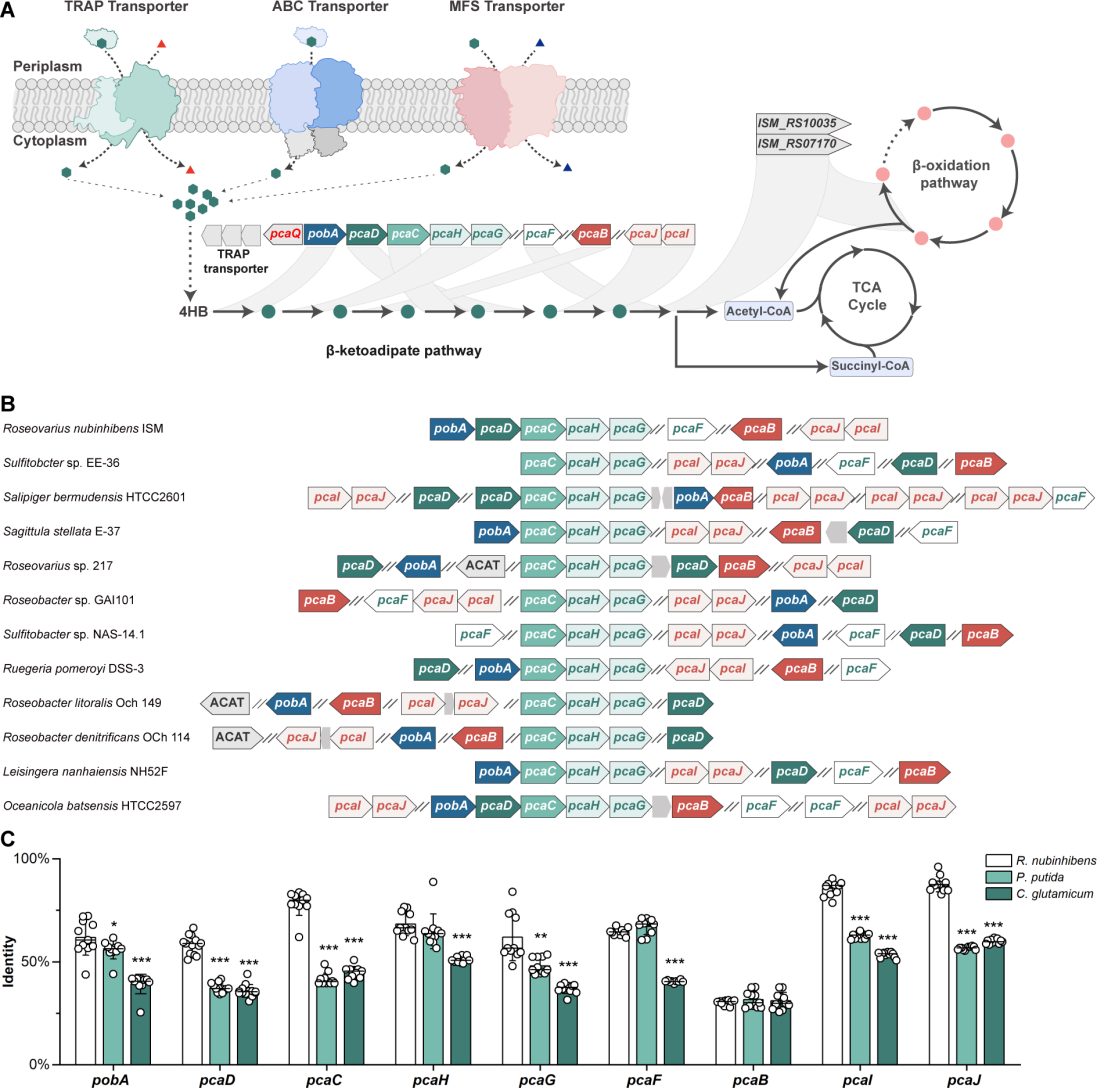
Illustration of the β-ketoadipate pathway in marine *Roseobacte*r clade. (**A**) 4HB uptake and metabolism in *R. nubinhibens*. The circles in green and pink represent the metabolic intermediates of β-ketoadipate and β-oxidation pathway, respectively. The genes are connected to the corresponding metabolic reactions. The genes *ISM_RS10035* and *ISM_RS07170* can catalyze the reactions in both β-ketoadipate and β-oxidation pathway, achieving the pathway-crosstalk. (**B**) Prediction of the β-ketoadipate pathway in eleven marine *Roseobacter* clade bacteria using *R. nubinhibens* as a reference. For simplification, acetyl-CoA C-acetyltransferase and acetyl-CoA C-acyltransferase are collectively termed ACAT, and the details of the genes and enzymes are listed in the Table S5. (**C**) Comparison of sequence identities of the predicted genes in the β-ketoadipate pathway of marine *Roseobacter* bacteria, using *R. nubinhibens* (white), *P. putida* KT2440 (light green), and *C. glutamicum* ATCC 13032 (dark green) as the references. Statistical significance was determined using a *t-*test (*P < 0.05; **P < 0.01; ***P < 0.001).

Successfully deciphering biological mechanisms relies on high-quality sequencing data and authentic references. Lacking thoroughly interrogated marine references, the analysis of marine microbes often depends on phylogenetically distant strains. For instance, we had to rely on *P. putida* and *C. glutamicum*, which have well-documented genomics and experimental data, to identify the missing genes, *pcaB* and *pcaIJ*. Now, we have comprehensively deciphered the degradation pathway of aromatic compounds, from uptake to entry into the central metabolism (Fig. 7A). With such findings, *R. nubinhibens* can serve as a novel reference for, but not limited to, the interrogation of aromatic metabolisms in marine bacteria. As a proof of concept, we successfully identified the essential genes involved in aromatic compound metabolism in eleven marine *Roseobacter* bacteria (Fig. 7B), and significantly higher sequence identities of the predicted genes were found compared to those determined using *P. putida* and *C. glutamicum* as references (Fig. 7C; Table S4). Based on our findings, we can also hypothesize that *Roseovarius* sp. 217, *Roseobacter denitrificans* OCh 114, and *Roseobacter litoralis* Och 149 (21) might utilize acetyl-CoA C-acetyltransferase or acetyl-CoA C-acyltransferase to accomplish aromatic degradation under *pcaF-*deficient genetic backgrounds (Fig. 7B).

## DISCUSSION

In the present study, we report a complete degradation pathway of 4HB in *R. nubinhibens*. Particularly, 4HB enters the cell via TRAP, ABC, and MFS transporters and is then degraded through the β-ketoadipate pathway with five genes located in the *pca* cluster, as well as *pcaF*, *pcaB,* and *pcaIJ* on separated loci in the genome. We identified the final thiolation as an intersection step which can be catalyzed by the 3-oxoadipyl-CoA thiolase from β-ketoadipate pathway and the acetyl-CoA C-acetyltransferase and acetyl-CoA C-acyltransferase from β-oxidation pathway (Fig. 7A). The aromatic compounds are eventually directed into central metabolism through this distinct pathway crosstalk, while similar but different pathway interaction for aromatics degradation has been reported in *P. putida* and *Escherichia coli* between β-ketoadipate and phenylacetate pathways but not β-oxidation (39). The spatial arrangement of different genes and the pathway crosstalk might exhibit adaptive evolutionary advantages in response to the highly oligotrophic and fluctuating ocean environments (18).

We highlight three main implications here. First, we identify the missing steps in the *Roseobacter* clade–mediated metabolism of aromatics in the ocean. These compounds represent a significant portion of oceanic carbon, originating from algal biomass, oil spills, and terrestrial runoffs (40–42). The natural degradation of these chemicals is essential for understanding the oceanic carbon cycle and advancing the bioremediation of aromatic pollutants. Actually, *Roseobacter* clade bacteria are not only abundant in the ocean but also in the estuaries and saline lakes (13, 43), where they exhibit increasingly important yet uncovered roles in the environment. Second, we provide a solid foundation with systems and genetic level evidence for aromatic metabolism in *Roseobacter* clade bacteria. While open questions remain concerning the degradation competency and pathways of more complex aromatics, such as coumaric acid and ferulic acid, this study provides a paradigm for future research through combined omics and genetic perturbation approaches. Indeed, *R. nubinhibens* is currently the only strain in marine *Roseobacter* clade bacteria with accessible CRISPR-Cas genome editing and regulation toolkits (20, 22), making it an emerging marine platform for biological investigation in complementary to other representative marine bacteria.

Finally, we propose a novel marine chassis for biological innovations. *Roseobacter* clade bacteria have the potential of degrading diverse aromatic compounds, which may originate from lignin biomass and waste streams, such as wastewater, through the β-ketoadipate pathway and TCA cycle. Beyond degradation, aromatics can be upcycled into value-added chemicals using protocatechuate, ketoadipate, or *cis,cis*-muconate as platform intermediates (44–48). Enhanced degradation could be achieved via approaches like bioaugmentation, while upcycling can be realized by rationally reprogramming *Roseobacter* clade bacteria using synthetic biology strategies (20). Notably, marine bacteria often exhibit superior capacities in adapting to inhibitors and stresses due to the relatively harsh conditions of the ocean (49), making them promising chassis for environmental and industrial applications. To conclude, our work not only expands the understanding of aromatic metabolism in marine bacteria but also highlights the potential of *Roseobacter* clade bacteria in mitigating aromatic pollution and driving biomanufacturing innovations.

## MATERIALS AND METHODS

### Strains and media

All strains used in this study are listed in Table S2. *E. coli* DH5α (Takara Bio) was employed for molecular cloning and was cultivated at 37°C in LB medium (10 g/L sodium chloride, 10 g/L tryptone, and 5 g/L yeast extract) or on solid LB plate with 1.5% agar. When necessary, gentamicin (20 µg/mL) or ampicillin (100 µg/mL) was added to the medium for selection and maintenance of plasmids. *R. nubinhibens* ISM (DSM 15170) served as the wild-type strain and was cultivated in marine broth 2216 (MB2216) medium at 30°C and 180 rpm or on solid MB2216 plate (1.5% agar). Gentamicin (20 µg/mL) was used to select transformants of *R. nubinhibens*. Growth experiments of *R. nubinhibens* were conducted in MBM containing 200 mM NaCl, 50 mM MgSO_4_, 10 mM NH_4_Cl, 10 mM KCl, 10 mM CaCl_2_, 1 mM K_2_HPO_4_, 0.1 mM FeEDTA, 0.1% vitamin, and 0.05% yeast extract (20).

### Transcriptomics, proteomics, and metabolomics

The wild-type *R. nubinhibens* strain was cultivated in MBM with 4HB and pyruvate as carbon sources, respectively, until the OD_600_ reached 1.0. Subsequently, 40 mL of culture was harvested via centrifugation at 4°C and 5,000 rpm for 10 min. The cell pellets were washed and resuspended with pre-cooled phosphate-buffered saline (PBS), frozen in liquid nitrogen for 5 min, and stored at −80°C until omics analysis.

For transcriptomic analysis, RNA sequencing (RNA-seq) library was prepared using the VAHTS Universal V6 RNA-seq Library Prep Kit, followed by sequencing on the DNBSEQ-T7 to generate the raw reads. Then, the raw data were quality-filtered using Trimmomatic to obtain high-quality clean reads. Next, the reads were then aligned to the *R. nubinhibens* ISM reference genome ASM15262v1 (RefSeq: GCF_000152625.1) using Rockhopper2. Gene expression quantification and normalization were performed with DESeq2 software. The significance of differential expression was performed using a negative binomial distribution test. Gene function was annotated by querying Gene Ontology (GO) and KEGG databases (50, 51). DEGs were identified based on *P*-value < 0.05 and fold change >2 or <0.5, followed by KEGG enrichment analysis.

The proteomics analysis was performed using the Q Exactive HF mass spectrometer (Thermo Fisher Scientific) coupled with an Easy-nLC 1200 HPLC system (Thermo Fisher Scientific). A total of 106,391 out of 388,675 mass spectra matched 38,418 peptides, leading to the identification of 4,396 high-confidence proteins. Data were processed with Proteome Discoverer 2.4.1.15 (Thermo Fisher Scientific) against the UniprotKB *R. nubinhibens* ISM database. Functional annotation of proteins was performed using the GO and KEGG databases. DEPs were identified with the thresholds of fold change >1.5 or <0.67 and *P*-value < 0.05. Spearman correlation analysis of DEPs was performed using R 4.1.1, followed by co-occurrence network generation with Gephi software.

For metabolomics, the metabolites were profiled using a Waters ACQUITY UPLC I-Class plus (Waters Corporation) fitted with a Thermo QE HF mass spectrometer (Thermo Fisher Scientific). An ACQUITY UPLC HSS T3 column was employed in both positive and negative modes. Then, the mass spectrometry data were processed using Progenesis QI v3.0 software (Nonlinear Dynamics). Metabolites were identified based on multiple dimensions, including retention time, secondary fragments, and isotopic distribution, and were annotated using the Human Metabolome Database (HMDB), Lipidmaps (v2.3), METLIN, and LuMet-Animal 3.0 databases. A total of 2,817 metabolites were identified. DEMs were identified with *P*-values < 0.05 and fold change >2 or <0.5.

### Plasmid construction

All plasmids, primers, and gRNA sequences used in this study are listed in Table S6 to S8. To generate the plasmid, DNA fragments were amplified using PrimerSTAR Max DNA Polymerase (Takara Bio) and assembled into the vector via the In-Fusion Snap Assembly Premix Kit (Takara Bio). All plasmids were extracted using the QIAprep Spin Miniprep Kit (Qiagen) and verified by Sanger sequencing and gel electrophoresis.

To construct the base editing plasmid pBeSpRYr for genome editing in *R. nubinhibens*, the DNA fragment containing *lacI-P_trc_*-inducible system, dSpRY, deaminase PmCDA1, and uracil glycosylase inhibitor (UGI) was amplified from plasmid pBeSpRY, constructed in our previous study (35), and then inserted into the plasmid pBBR1MCS-5 via In-Fusion assembly. The 20 bp spacers of gRNA cassette targeting the specific genome locus were constructed on the pTemplate plasmid via inverse PCR, and then, the entire gRNA cassette was assembled on the pBeSpRYr via DNA assembly approach, thereby generating pBeSpRYr-01 (with gRNA01) and pBeSpRYr-02 (with gRNA02). Based on the base editing plasmid pWY for *R. nubinhibens* in our previous study (22), we constructed the pWY-hw01 (with gRNA04) and pWY-hw02 (with gRNA05) by embedding the specific gRNA cassettes.

### Transformation of *R. nubinhibens*

The working plasmids were transformed into *R. nubinhibens* via electroporation. To prepare the electrocompetent cells, *R. nubinhibens* was grown in MB2216 medium until the OD_600_ reached 0.5. Then, 30 mL of culture was harvested by centrifugation at 5,000 rpm and 4°C for 10 min, followed by two washes with pre-cooled buffered sucrose solution (272 mM sucrose, 7 mM Na_3_PO_4_, and 1 mM MgCl_2_). The cells were then resuspended and aliquoted into 80 µL portions, which were stored at −80°C until use. The competent cells and the working plasmid were mixed and transferred in a 0.1 cm MicroPulser Electroporation Cuvette (Bio-Rad), and the electroporation was conducted using MicroPulser Electroporator (Bio-Rad) (1.8 kV), after which the cells were immediately recovered in fresh MB2216 medium. The recovered cells were induced in liquid medium with gentamicin (20 µg/mL) and IPTG (0.5 mM) for 12 h before being plated on the selective plate (22). Single colonies were randomly selected for colony PCR and sequencing of the target sites to confirm successful genome editing.

### Plasmid curing

To cure the working plasmids, the edited strains were cultivated in MB2216 medium without antibiotics for two rounds of transfer and then streaked onto non-selective plates to obtain the single colonies. The colonies were randomly selected to check the existence of plasmids by colony PCR and the antibiotic resistance. When the colony PCR showed no signal for the plasmid and the edited strains could not grow in the medium with gentamicin, the plasmid was regarded as cured.

### Quantification of 4HB and pyruvate

For the quantification of 4HB and pyruvate, the samples were centrifuged at 12,000 rpm for 1 min. The supernatant was filtered through 0.22 µm membrane and then diluted 10-fold with double-distilled water. The concentrations of 4HB and pyruvate were determined using an Agilent 1260 HPLC system equipped with a UV detector (Agilent Technology) at 210 nm, with an injection volume of 10 µL. Chromatographic separation was achieved using an EC-C18 column (Agilent Technologies) with 10% acetonitrile and 90% formic acid (0.1% concentration) as the mobile phase at a flow rate of 0.8 mL/min.

### Verification of transcriptome and quantitative real-time PCR analysis

The total RNA samples used for RNA-seq were reverse-transcribed into cDNA using PrimeScript RT Reagent Kit with gDNA Easer (Perfect Real Time; Takara Bio). Two target genes, *ISM_RS04035* (*pcaF*) and *ISM_RS10035*, were selected for RT-qPCR to validate the RNA-seq data. RT-qPCR was performed using TB Green Premix Ex Taq II (Tli RNaseH Plus; Takara Bio) on Applied Biosystems QuantStudio 5 system (Thermo Fisher Scientific). To analyze the gene expressions in the edited strains, total RNA was extracted using the RNAprep Pure Cell/Bacteria Kit (Tiangen), followed by reverse transcription and RT-qPCR performed according to the aforementioned methods. The gene *ISM_RS12040*, encoding for the 16S ribosomal RNA, was selected as the housekeeping gene. All primers used for qPCR are given in Table S7.

### Bioinformatic analysis

All results of sequence identity in this study were obtained by aligning amino acid sequences. All amino acid sequences were downloaded from NCBI and listed in Table S9. The sequences were aligned using the ClustalW algorithm integrated in MEGA11 (52), and then, the alignment results were exported in FASTA format. Subsequently, the FASTA file was processed using the ESPript 3.0 (https://espript.ibcp.fr) to generate sequence alignment maps (53).

The Basic Local Alignment Search Tool (BLAST) was used to perform homology analysis. To identify homologous genes and determine sequence identity, the amino acid sequence of the target gene from reference strains was queried against the protein databases of 11 specified marine *Roseobacter* bacteria using blastp (https://blast.ncbi.nlm.nih.gov/Blast.cgi?PROGRAM=blastp&PAGE_TYPE=BlastSearch&LINK_LOC=blasthome). The results provided both the identification of homologous genes in each of the 11 marine *Roseobacter* bacteria and their corresponding percentage identity values relative to the query sequence.

## Data Availability

The transcriptomic sequencing data have been deposited in the National Center for Biotechnology Information (NCBI) database under the accession number PRJNA1234464. The mass spectrometry proteomics data have been deposited to the ProteomeXchange Consortium via the iProX partner repository with the datasetdata set identifier PXD061672. The metabolomics data have been deposited to the MetaboLights repository with the study identifier MTBLS12306.

## SUPPLEMENTAL MATERIAL

Supplemental material aem.00978-25-s0001.pdf Tables S1 to S9; Figures S1 to S11.

ASM does not own the copyrights to Supplemental Material that may be linked to, or accessed through, an article. The authors have granted ASM a non-exclusive, world-wide license to publish the Supplemental Material files. Please contact the corresponding author directly for reuse.
