# Supplementary material for: Pathway crosstalk enables degradation of aromatic compounds in marine Roseobacter clade bacteria
Source: Appl Environ Microbiol. 2025 Aug 12;91(9):e00978-25. doi: 10.1128/aem.00978-25 (PMC12442348; doi:10.1128/aem.00978-25)
Supplement: Supplemental material — Tables S1 to S9; Figures S1 to S11. [file aem.00978-25-s0001.pdf]

Supplemental Material

Pathway crosstalk enables degradation of aromatic compounds in marine *Roseobacter* clade bacteria

Huan-Wei Xu [1], Xiao-Yan Wang [1], Ying Wei [1], Yi-qi Cao [2,3], Shu-Guang Wang [1,4,5], Peng-Fei Xia [1,*]

[1] School of Environmental Science and Engineering, Shandong University, Qingdao, China

[2] Northern Region Persistent Organic Pollution Control (NRPOP) Laboratory, Faculty of Engineering and Applied Science, Memorial University, St John's, Canada

[3] School of Marine Sciences, Ningbo University, Ningbo, China

[4] Sino-French Research Institute for Ecology and Environment, Shandong University, Qingdao, China

[5] Weihai Research Institute of Industrial Technology, Shandong University, Weihai, China

* Correspondence:

Peng-Fei Xia

School of Environmental Science and Engineering, Shandong University, Qingdao, China. Email: pfxia@sdu.edu.cn

Table S1. Genes with significant changes in the TCA cycle at the protein level.

Genes	Polypeptides	Fold change	p-value
sucC	ADP-forming succinate--CoA ligase subunit beta	1.60	9.539E-05
sucD	succinate--CoA ligase subunit alpha	1.71	0.0002063

Table S2. Strains used in this study.

Name	Features	Source
E. coli DH5α	Commercial *E. coli* strain for molecular cloning	Takara Bio. Tech.
R. nubinhibens ISM	Wild type strain (DSM 15170)	China General Microbiological Culture Collection Center
HW01	Wild type, *ISM_RS06170* Gln119*	This study
HW02	Wild type, *ISM_RS03130* Gln67*	This study
HW04	Wild type, *pcaF* Gln181*	This study
HW05	Wild type, *ISM_RS10035* Gln174*	This study
HW06	Wild type, *pcaF* Gln181*, *ISM_RS10035* Gln174*	This study

Table S3. Genes encoding key enzymes involved in β-oxidation pathway.

**Enzymes**	**EC number**	**Genes**
Acetyl-CoA C-acyltransferase	EC 2.3.1.16	*ISM_RS10035*
		ISM_RS07170
Acetyl-CoA C-acetyltransferase	EC 2.3.1.9	*ISM_RS09365*
		ISM_RS02035

Table S4. The accession ID and identity of the predicted genes.

Genes	Predicted genes	Identity to *P. putida*	Identity to *C. glutamicum*	Identity to *R. nubinhibens*
***Sulfitobacter* sp. EE-36 (GCF_000152605.1)** [a]				
pobA	*EE36_RS08120*	54.73%	41.43%	56.56%
pcaD	*EE36_RS03205*	34.10%	35.74%	58.02%
pcaC	*EE36_RS10975*	39.50%	45.45%	83.61%
pcaH	*EE36_RS10970*	88.68%	53.18%	66.38%
pcaG	*EE36_RS10965*	44.28%	38.24%	56.59%
pcaF	*EE36_RS06315*	71.11%	40.29%	62.88%
pcaB	*EE36_RS03215*	33.56%	32.97%	31.20%
pcaI	*EE36_RS09460*	61.14%	54.66%	87.77%
pcaJ	*EE36_RS09455*	56.73%	61.65%	87.50%
***Salipiger bermudensis* HTCC2601 (GCF_000153725.1)**				
pobA	*R2601_RS05405*	54.48%	40.47%	58.10%
pcaD	*R2601_RS05375*	37.05%	39.08%	66.41%
	R2601_RS04930	37.12%	30.80%	53.82%
pcaC	*R2601_RS05380*	45.38%	50.83%	77.05%
pcaH	*R2601_RS05385*	62.23%	50.91%	76.37%
pcaG	*R2601_RS05390*	52.44%	31.39%	73.91%
pcaF	*R2601_RS22925*	69.33%	40.49%	64.68%
pcaB	*R2601_RS05410*	37.71%	33.46%	29.89%
pcaI	*R2601_RS04630*	37.98%	38.50%	40.48%
	R2601_RS10000	63.91%	53.21%	78.60%
	R2601_RS12160	43.06%	42.80%	44.78%
	R2601_RS22915	41.18%	41.46%	43.50%
pcaJ	*R2601_RS04635*	49.53%	47.55%	46.08%
	R2601_RS10005	55.29%	59.71%	87.50%
	R2601_RS12165	45.67%	51.47%	53.40%
	R2601_RS22920	46.51%	48.53%	52.24%
***Sagittula stellate* E-37 (GCF_000169415.1)**				
pobA	*SSE37_RS00395*	56.73%	38.54%	60.46%
pcaD	*SSE37_RS14975*	35.61%	34.41%	53.12%

pcaC	*SSE37_RS00400*	40.34%	46.25%	77.87%
pcaH	*SSE37_RS00405*	61.90%	49.55%	62.13%
pcaG	*SSE37_RS00410*	45.13%	37.56%	55.28%
pcaF	*SSE37_RS22960*	60.85%	39.23%	65.25%
pcaB	*SSE37_RS14965*	37.57%	37.5%	28.33%
pcaI	*SSE37_RS05530*	63.32%	53.66%	82.61%
pcaJ	*SSE37_RS05535*	57.24%	59.22%	82.69%
***Roseovarius* sp. 217 (GCF_000152845.1)**				
pobA	*ROS217_RS11090*	44.00%	25.44%	43.75%
pcaD	*ROS217_RS11480*	34.96%	44.00%	50.95%
	ROS217_RS09690	36.95%	33.19%	52.42%
pcaC	*ROS217_RS09710*	42.02%	46.28%	81.97%
pcaH	*ROS217_RS09705*	66.08%	50.68%	76.57%
pcaG	*ROS217_RS09700*	52.26%	35.86%	73.30%
pcaF	-	-	-	-
pcaB	*ROS217_RS09685*	33.72%	37.5%	27.79%
pcaI	*ROS217_RS00950*	62.61%	54.25%	90.39%
pcaJ	*ROS217_RS00955*	55.77%	58.74%	92.31%
***Roseobacter* sp. GAI101 (GCF_000156335.1)**				
pobA	*RGAI101_RS13955*	56.01%	41.25%	57.58%
pcaD	*RGAI101_RS16675*	36.65%	34.94%	58.78%
pcaC	*RGAI101_RS07430*	40.50%	46.22%	82.64%
pcaH	*RGAI101_RS07435*	64.96%	52.27%	65.11%
pcaG	*RGAI101_RS07440*	43.72%	39.59%	53.66%
pcaF	*RGAI101_RS06470*	68.58%	40.53%	67.50%
pcaB	*RGAI101_RS00255*	28.54%	26.32%	30.69%
pcaI	*RGAI101_RS09395*	61.14%	54.66%	87.34%
	RGAI101_RS06480	43.06%	40.66%	45.09%
pcaJ	*RGAI101_RS09400*	57.21%	60.68%	96.06%
	RGAI101_RS06475	46.26%	49.76%	48.76%
***Sulfitobacter* sp. NAS-14.1 (GCF_000152645.1)**				
pobA	*NAS141_RS11035*	54.99%	41.69%	56.30%

pcaD	*NAS141_RS06080*	34.66%	35.74%	57.63%
pcaC	*NAS141_RS14175*	39.50%	45.45%	82.79%
pcaH	*NAS141_RS14170*	63.68%	53.18%	66.38%
pcaG	*NAS141_RS14165*	44.28%	38.24%	56.59%
pcaF	*NAS141_RS17715*	68.08%	40.24%	63.84%
	NAS141_RS09200	71.11%	40.29%	62.88%
pcaB	*NAS141_RS05180*	28.54%	25.56%	31.25%
pcaI	*NAS141_RS12455*	61.14%	54.07%	87.72%
pcaJ	*NAS141_RS12450*	56.73%	61.17%	87.02%
***Ruegeria pomeroyi* DSS-3 (GCF_000011965.2)**				
pobA	*SPO_RS20005*	57.54%	38.32%	69.67%
pcaD	*SPO_RS22025*	41.63%	36.95%	62.84%
pcaC	*SPO_RS20000*	41.67%	44.63%	79.84%
pcaH	*SPO_RS19995*	63.79%	50.00%	73.03%
pcaG	*SPO_RS19990*	53.47%	36.68%	83.98%
pcaF	*SPO_RS03825*	70.18%	41.08%	61.65%
pcaB	*SPO_RS11685*	27.74%	29.76%	30.69%
pcaI	*SPO_RS15615*	62.88%	53.88%	83.77%
pcaJ	*SPO_RS15620*	57.69%	58.25%	84.62%
***Roseobacter litoralis* Och 149 (GCF_000154785.2)**				
pobA	*RLO149_RS10480*	57.14%	41.30%	67.87%
pcaD	*RLO149_RS02470*	38.52%	34.91%	60.31%
pcaC	*RLO149_RS03305*	39.50%	42.98%	77.87%
pcaH	*RLO149_RS03300*	59.40%	50.68%	65.52%
pcaG	*RLO149_RS03295*	47.26%	38.58%	54.55%
pcaF	-	-	-	-
pcaB	*RLO149_RS09695*	27.38%	29.35%	30.58%
pcaI	*RLO149_RS06115*	63.04%	51.63%	86.58%
pcaJ	*RLO149_RS06105*	56.73%	59.71%	88.46%
***Roseobacter denitrificans* OCh 114 (GCF_000014045.1)**				
pobA	*RD1_RS10760*	56.23%	40.47%	64.87%
pcaD	*RD1_RS18005*	36.96%	35.34%	59.54%

pcaC	*RD1_RS17285*	39.50%	42.15%	77.05%
pcaH	*RD1_RS17290*	59.83%	50.68%	65.09%
pcaG	*RD1_RS17295*	46.11%	38.46%	54.55%
pcaF	-	-	-	-
pcaB	*RD1_RS11795*	27.61%	28.86%	30.45%
pcaI	*RD1_RS09840*	63.48%	52.03%	87.01%
pcaJ	*RD1_RS09830*	57.21%	60.19%	88.46%
***Leisingera nanhaiensis* NH52F (GCF_000473225.1)**				
pobA	*NANH_RS0120855*	59.08%	40.00%	72.24%
pcaD	*NANH_RS0116770*	40.47%	35.60%	63.36%
pcaC	*NANH_RS0120850*	39.50%	39.67%	80.80%
pcaH	*NANH_RS0120845*	64.76%	49.77%	74.48%
pcaG	*NANH_RS0120840*	52.26%	35.03%	71.36%
pcaF	*NANH_RS0115220*	60.50%	41.46%	64.23%
pcaB	*NANH_RS0104215*	30.10%	27.00%	31.19%
pcaI	*NANH_RS0118945*	65.07%	54.25%	81.22%
pcaJ	*NANH_RS0118940*	55.88%	60.29%	84.95%
***Oceanicola batsensis* HTCC2597 (GCF_000152725.1)**				
pobA	*OB2597_RS18540*	61.64%	41.71%	71.98%
pcaD	*OB2597_RS18535*	39.68%	34.69%	59.00%
pcaC	*OB2597_RS18530*	48.74%	41.32%	61.98%
pcaH	*OB2597_RS18525*	56.54%	49.09%	60.34%
pcaG	*OB2597_RS18520*	46.00%	36.04%	47.85%
pcaF	*OB2597_RS14805*	64.16%	40.44%	66.67%
	OB2597_RS03890	67.83%	40.24%	64.59%
pcaB	*OB2597_RS18510*	34.41%	32.49%	29.76%
pcaI	*OB2597_RS02335*	42.59%	42.80%	42.49%
	OB2597_RS19635	60.70%	50.61%	88.16%
pcaJ	*OB2597_RS02330*	40.58%	47.78%	51.24%
	OB2597_RS19630	55.77%	58.74%	84.62%

[a] The content in brackets indicates the NCBI RefSeq assembly accession number.

Table S5. Genes encoding key enzyme involved in pathway crosstalk.

Genes	Encoding enzymes
Roseobacter denitrificans **OCh 114 (GCF_000014045.1)** [a]	
RD1_RS15580	acetyl-CoA C-acetyltransferase
RD1_RS18180	acetyl-CoA C-acetyltransferase
RD1_RS00925	acetyl-CoA C-acyltransferase family protein
Roseobacter litoralis **Och 149 (GCF_000154785.2)**	
RLO149_RS21595	acetyl-CoA C-acyltransferase family protein
RLO149_RS13385	acetyl-CoA C-acetyltransferase
RLO149_RS02295	acetyl-CoA C-acetyltransferase
RLO149_RS18990	acetyl-CoA C-acetyltransferase
Roseovarius **sp. 217 (GCF_000152845.1)**	
ROS217_RS05060	acetyl-CoA C-acyltransferase
ROS217_RS09915	acetyl-CoA C-acyltransferase

[a] The content in brackets indicates the NCBI RefSeq assembly accession number.

Table S6. Plasmids used in this study.

Name	Description	Source
pTemplate	*pUC ori*, gRNA scaffold, *bla*	Lab stock
pBeSpRY	*oriR 101*, *bla*, *lacI*-P_{trc}, dSpRY-PmCDA1-ugi	Lab stock (1)
pWY	pBBR1MCS-5, *GmR*, *lacI*-P_{trc}, dCas9-PmCDA1-ugi	Lab stock (2)
pBeSpRYr	pBBR1MCS-5, *GmR*, *lacI*-P_{trc}, dSpRY-PmCDA1-ugi	This study
pgRNA01	pTemplate, gRNA01	This study
pgRNA02	pTemplate, gRNA02	This study
pgRNA04	pTemplate, gRNA04	This study
pgRNA05	pTemplate, gRNA05	This study
pBeSpRYr-01	pBeSpRYr, gRNA01	This study
pBeSpRYr-02	pBeSpRYr, gRNA02	This study
pWY-hw01	pWY, gRNA04	This study
pWY-hw02	pWY, gRNA05	This study

Table S7. Primers used in this study.

Primer	Sequence
**Primers for inverse PCR**	
XIA-XHW-92	CGATCAGGATGCCTTTGCGCGTTTTAGAGCTAGAAATAGC
XIA-XHW-93	GCGCAAAGGCATCCTGATCGGCTAGCATTATACCTAGGAC
XIA-XHW-94	GCGCAGGACGCCTATGCGATGTTTTAGAGCTAGAAATAGC
XIA-XHW-95	ATCGCATAGGCGTCCTGCGCGCTAGCATTATACCTAGGAC
XIA-XHW-142	GCAGATGAAGGACGCCTATGGTTTTAGAGCTAGAAATAGC
XIA-XHW-143	CATAGGCGTCCTTCATCTGCGCTAGCATTATACCTAGGAC
XIA-XHW-150	GCAGACGCGGCAGGTGAAGAGTTTTAGAGCTAGAAATAGC
XIA-XHW-151	TCTTCACCTGCCGCGTCTGCGCTAGCATTATACCTAGGAC
**Primers for In-Fusion DNA assembly**	
XIA-XHW-96	TTGACTACCGGAAGCAGTGTTCTAGATTGTAAAACGACGGCCAGTC
XIA-XHW-97	CATTTGAGAAGCACACGGTCACAGGAAACAGCTATGACCG
XIA-XHW-98	GACTGGCCGTCGTTTTACAATCTAGAACACTGCTTCCGGTAGTCAA
XIA-XHW-99	CGGTCATAGCTGTTTCCTGTGACCGTGTGCTTCTCAAATG
XIA-XHW-131	TGGCTGGCTGGCATAAATATCTCACTCGCAATCAAATTCA
XIA-XHW-132	AAGCACACGGTCACAGGAAACAGCTATGACCGTCTCGGTT
XIA-XHW-133	TGAATTTGATTGCGAGTGAGATATTTATGCCAGCCAGCCA
XIA-XHW-134	AACCGAGACGGTCATAGCTGTTTCCTGTGACCGTGTGCTT
XIA-XHW-140	AGCGGATTTGAACGTTGCGAATCCTTGACAGCTAGCTCAG
XIA-XHW-141	CTGAGCTAGCTGTCAAGGATTCGCAACGTTCAAATCCGCT
**Primers for colony PCR and sequencing**	
XIA-XHW-100	ACCACGCTCAACAAGATGTG
XIA-XHW-101	TTCGTTCACCTCCCACAGAT
XIA-XHW-102	CGAAGTGGTCGATCACATGT
XIA-XHW-103	ACTATATCCGGACGCCGATT
XIA-XHW-104	ATGCTCGTCGCGTTCCACCT
XIA-XHW-106	TTTGTGAACCCGGCGATCGA
XIA-XHW-107	TTAGGTGGCGGTACTTGGGT
XIA-XHW-108	GCAGTCGCCCTAAAACAAAG
XIA-XHW-144	CGATTCCGTCCTTCTCAAGA

XIA-XHW-145	TCCATGTCATGCGCCTTGGA
XIA-XHW-146	TCTCCTCGCTGTGACCAAAC
XIA-XHW-147	TTCGTGGGCAAGATCTATGG
XIA-XHW-148	GTCTTGCGGAAGATGGCGTT
XIA-XHW-149	TTTGGGCTTTGCGGCATTCC
**Primers for RT-qPCR**	
XIA-XHW-86	AAACGATGAATGCCAGTCGT
XIA-XHW-87	TGCGCGTTGCTTCGAATTAA
XIA-XHW-109	ATTGGCACAGCCAAGGATCA
XIA-XHW-110	GTCTGCGACTATATCCGGAC
XIA-XHW-111	GGCATGAAGGCCGCGATGAT
XIA-XHW-112	GTGATCGACCACTTCGCCAT
XIA-XHW-113	GCCCACACGAAATTTGGCAA
XIA-XHW-114	TTCCAGCCCCTGCTTGGAAA
XIA-XHW-115	TCAGGCGCATGTGAATGCAG
XIA-XHW-116	CATGTTTTCCTGACCACCGG
XIA-XHW-117	CACCCAGGTGATGGAACAGG
XIA-XHW-118	CGATATAGGCCTGACCGGCA

Table S8. gRNA sequences used in this study.

gRNA	Target	PAM	Protospacer
gRNA01	*ISM_RS06170*	ACG	GCAGATGAAGGACGCCTATG
gRNA02	*ISM_RS03130*	AGA	GCAGACGCGGCAGGTGAAGA
gRNA04	*ISM_RS04035* (*pcaF*)	TGG	CGATCAGGATGCCTTTGCGC
gRNA05	*ISM_RS10035*	CGG	GCGCAGGACGCCTATGCGAT

Table S9. NCBI accession number of the proteins encoded by genes for amino acid sequence alignment.

**Genes**	**Accession (Protein ID)**
P. putida **KT2440 (GCF_000007565.2)** [a]	
PP_RS07125 (pcaB)	WP_010952487.1
PP_RS16280 (pcaI)	WP_004374230.1
PP_RS16285 (pcaJ)	WP_010954014.1
C. glutamicum **ATCC 13032 (GCF_000011325.1)**	
Cgl2396 (pcaB)	WP_011015090.1
Cgl2390 (pcaI)	WP_003859254.1
Cgl2389 (pcaJ)	WP_003859257.1
R. nubinhibens **ISM (GCF_000152625.1)**	
ISM_RS06170	WP_040617899.1
ISM_RS03130	WP_009812656.1
ISM_RS03135	WP_009812657.1
ISM_RS04035 (pcaF)	WP_009812840.1
ISM_RS10035	WP_009814044.1

[a] The content in brackets indicates the NCBI RefSeq assembly accession number.

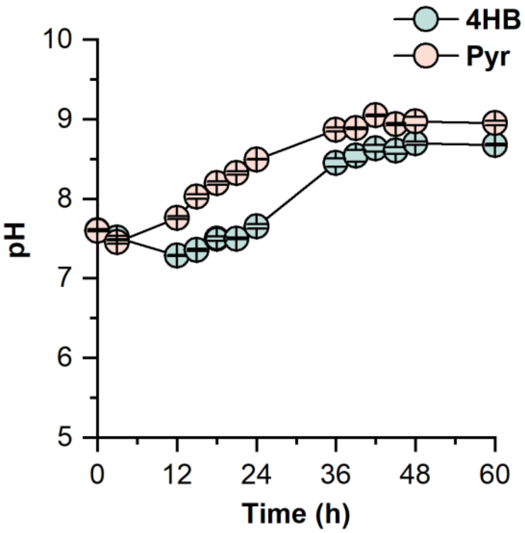

Figure S1. pH variations of *R. nubinhibens* growing in MBM with 4HB and pyruvate. The initial pH was adjusted to 7.5. The experiments were performed in triplicate, and error bars represent the standard deviations.

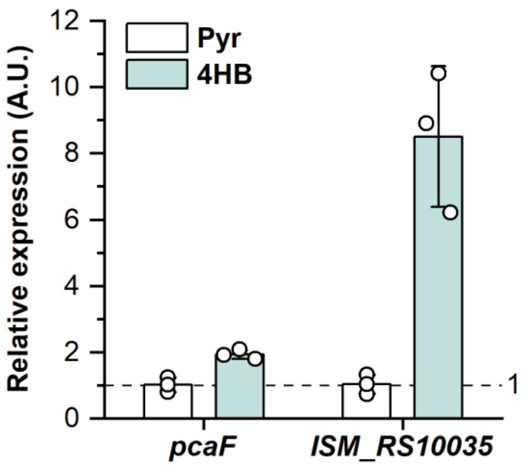

Figure S2. Relative expression levels of *pcaF* and *ISM_RS10035* in *R. nubinhibens* cultured with 4HB and pyruvate. The experiments were performed in triplicate, and error bars represent the standard deviations.

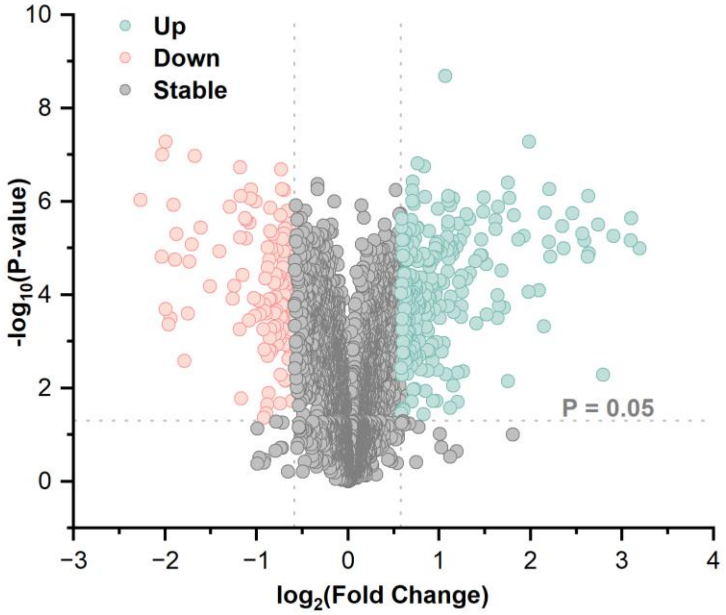

Figure S3. Volcano plot showing the fold changes in protein abundance. Up-regulated DEPs were identified with $\log_2$ (fold change) > 0.585 and p-value < 0.05 and highlighted in green. Down-regulated DEPs were identified with $\log_2$ (fold change) < -0.585 and p-value < 0.05 and highlighted in pink.

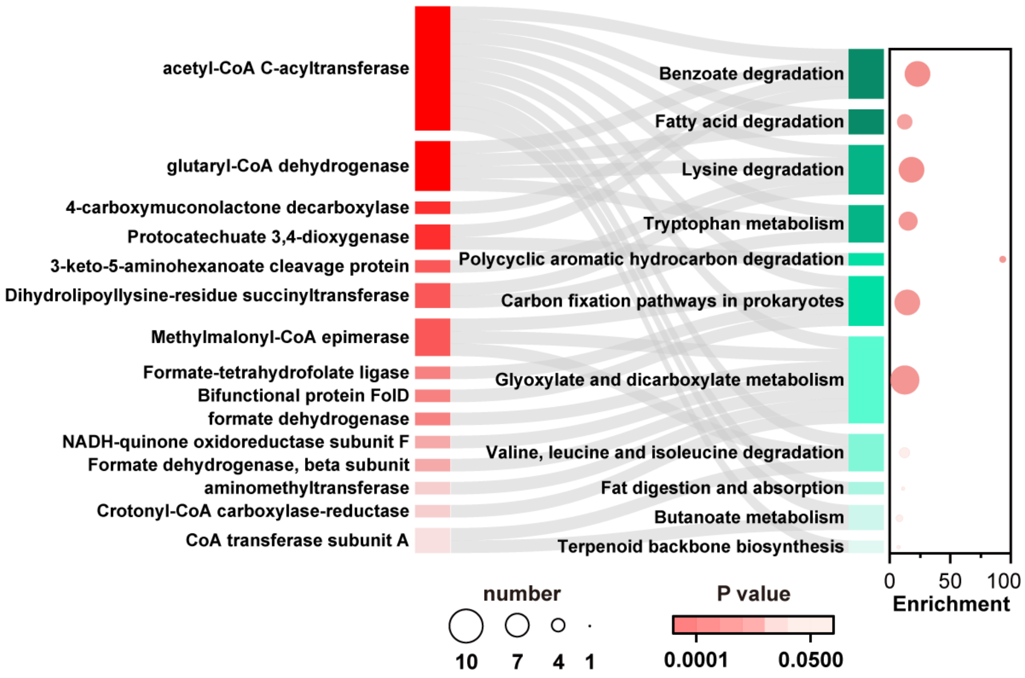

Figure S4. KEGG pathway enrichment analysis of DEPs. The circle size represents the number of enriched genes in each pathway, and the gradient red in the circle represents the p-value. Different shades of red and green rectangles indicate different proteins and metabolic pathways.

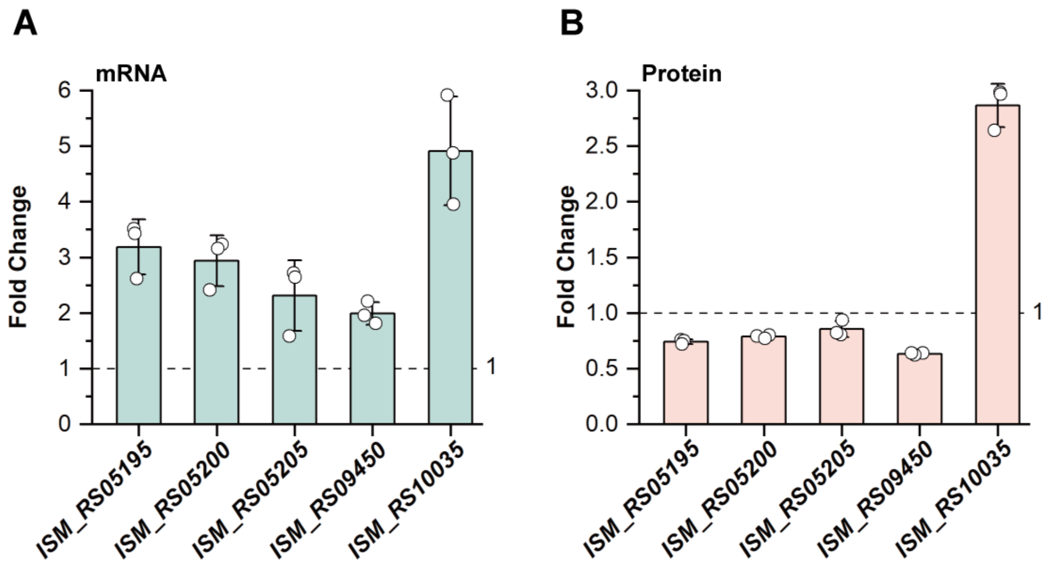

Figure S5. Fold changes of the DEGs enriched in pyruvate metabolism pathway at the **(A)** mRNA and **(B)** protein levels.

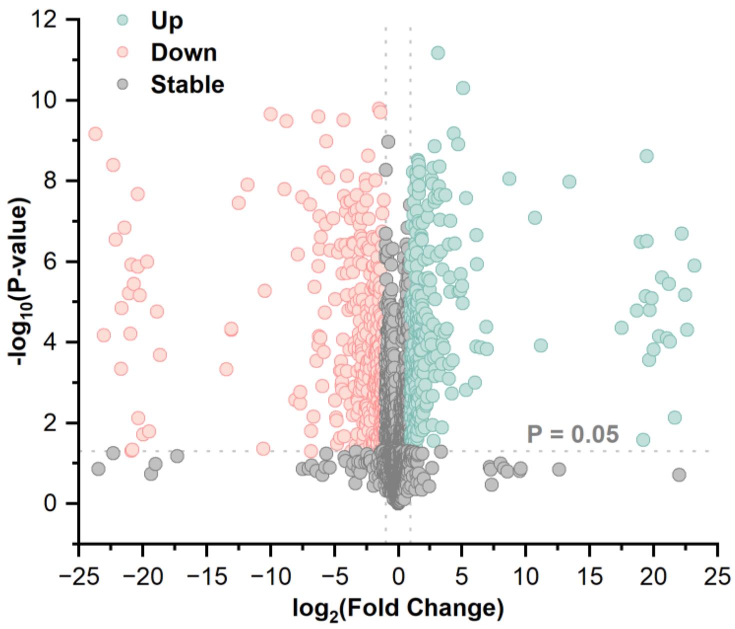

Figure S6. Volcano plot showing the fold changes in metabolites. Up-regulated DEMs were identified with $\log_2$ (fold change) > 1 and p-value < 0.05 and highlighted in green. Down-regulated DEMs were identified with $\log_2$ (fold change) < -1 and p-value < 0.05 and highlighted in pink.

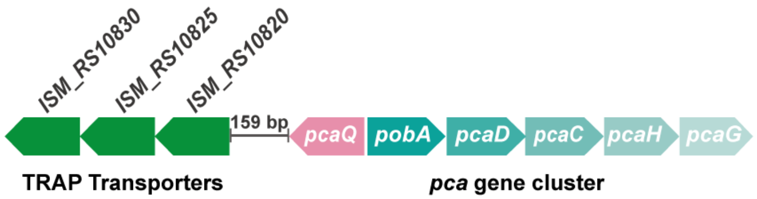

Figure S7. Spatial arrangement of TRAP transporter-associated genes and the *pca* gene cluster.

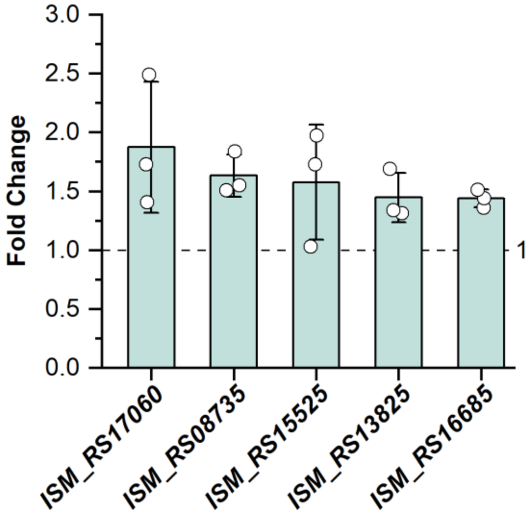

Figure S8. Fold changes of mRNAs of the genes encoding the proteins related to MFS transporters.

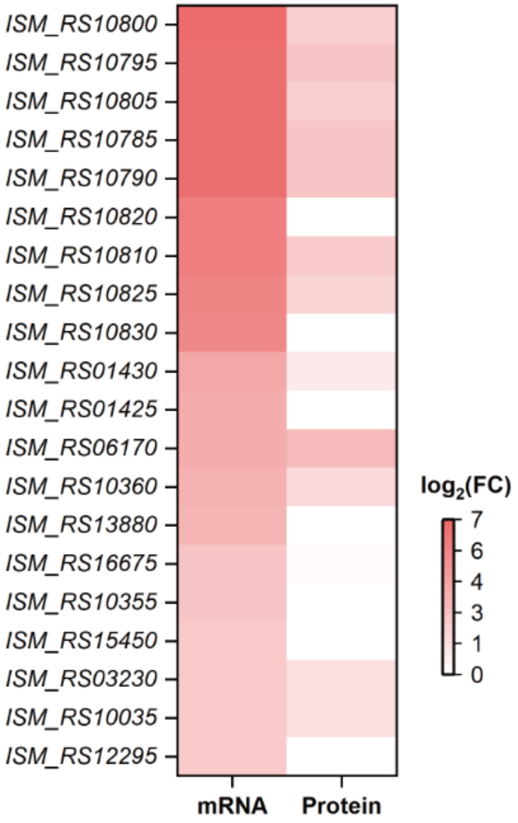

Figure S9. Fold changes of the top 20 DEGs at the mRNA and protein levels.

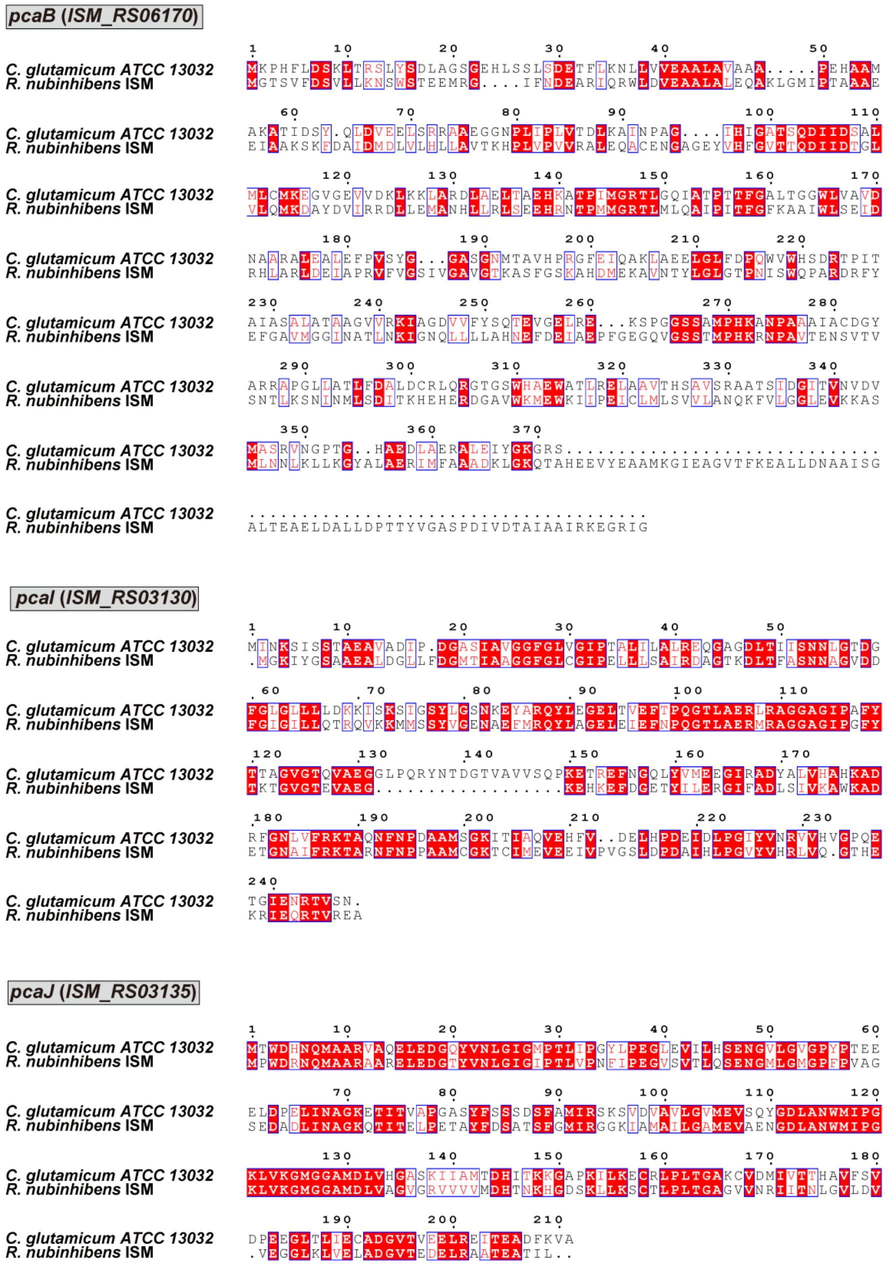

Figure S10. Visualization of alignments between amino acid sequences encoded by *ISM_RS06170*, *ISM_RS03130* and *ISM_RS03135* of *R. nubinhibens* and *pcaB*, *pcaI* and *pcaJ* of *C. glutamicum* ATCC 13032. White letters with red background indicate identical sequences. Red letters with white background highlight non-identical residues with similar physicochemical properties.

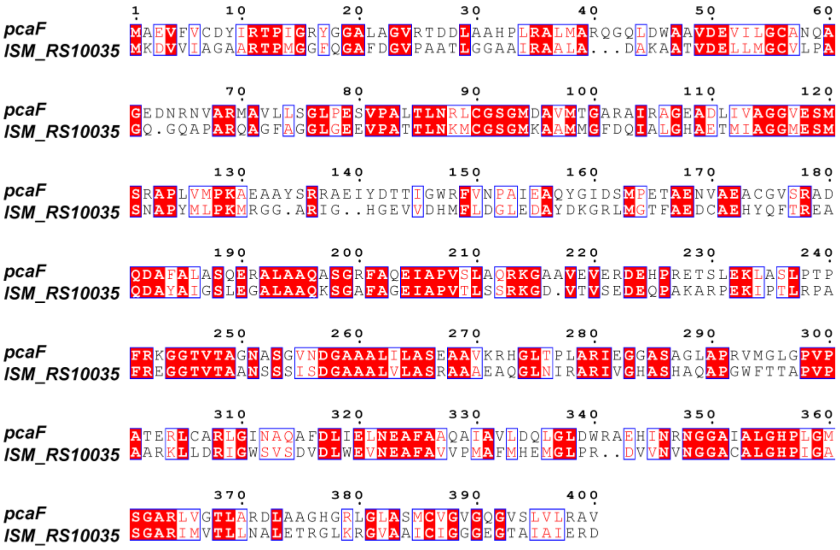

Figure S11. Visualization of alignment between amino acid sequences encoded by *pcaF* and *ISM_RS10035* in *R. nubinhibens*. White letters with red background indicate identical sequences. Red letters with white background highlight non-identical residues with similar physicochemical properties.

Reference

1. Li X, Wei Y, Wang S-Y, Wang S-G, Xia P-F. 2025. One-for-all gene inactivation via PAM-independent base editing in bacteria. J Biol Chem 301:108113. https://doi.org/10.1016/j.jbc.2024.108113.
2. Wei Y, Feng LJ, Yuan XZ, Wang SG, Xia PF. 2023. Developing a base editing system for marine *Roseobacter* clade bacteria. ACS Synth Biol 12:2178-2186. https://doi.org/10.1021/acssynbio.3c00259.
